# Comparison of astigmatism correction and visual outcomes in mix-and-match implantations of trifocal intraocular lenses with femtosecond laser-assisted arcuate keratotomy and contralateral bifocal Toric intraocular lenses

**DOI:** 10.3389/fmed.2023.1237319

**Published:** 2023-08-04

**Authors:** Jiying Shen, Zhixiang Hua, Limei Zhang, Baoxian Zhuo, Wenqian Shen, Xuanzhu Chen, Haike Guo, Jin Yang

**Affiliations:** ^1^Department of Ophthalmology, Shanghai Heping Eye Hospital, Shanghai, China; ^2^Department of Ophthalmology and the Eye Institute, Eye and Ear, Nose, and Throat Hospital, Fudan University, Shanghai, China; ^3^The Key Laboratory of Myopia, Ministry of Health, Shanghai, China; ^4^Shanghai Key Laboratory of Visual Impairment and Restoration, Shanghai, China; ^5^Key National Health Committee of the Key Laboratory of Myopia, Fudan University, Shanghai, China; ^6^The Key Laboratory of Myopia, Chinese Academy of Medical Sciences, Shanghai, China

**Keywords:** femtosecond laser-assisted arcuate keratotomy, mix-and-match, astigmatism correction, residual astigmatism, cataract surgery

## Abstract

**Introduction:**

Astigmatism reduces the postoperative visual performance after non-toric intraocular lenses (IOLs) implantation, and limits the use of refractive IOLs in cataract surgery. The purpose of this study was to compare the efficacy in astigmatism correction and the postoperative visual outcomes between the implantation of a trifocal IOL with femtosecond laser-assisted arcuate keratotomy (FSAK) in one eye and a bifocal toric IOL (TIOL) in the other, in patients with cataract and moderate astigmatism.

**Methods:**

This prospective observational paired-eye study enrolled patients with cataract and corneal astigmatism (CA) between 0.75 and 2.25 D in both eyes. The patients underwent a mix-and-match treatment comprising trifocal IOL implantation with FSAK and bifocal TIOL implantation. We compared the visual acuity (VA) at all distances, defocus curve, postoperative refractive astigmatism (RfA), CA, high-order aberrations, modulation transfer function (MTF) curve, and Strehl ratio between the two eye groups.

**Results:**

In total, 41 patients (82 eyes) were enrolled and completed a 6-month follow-up. The 1- and 3-month uncorrected distance VA and 3-month uncorrected near VA were greater in eyes with bifocal TIOLs than with trifocal IOLs and FSAK (*p* = 0.036, 0.010, and 0.030, respectively), whereas the latter had greater uncorrected intermediate VA at every visit and greater VA in the intermediate range of defocus curve (at −1.50 and − 2.00 D) than the eyes with bifocal TIOLs. The postoperative RA of the eyes with trifocal IOL and FSAK was significantly higher than that of the bifocal TIOL-implanted eyes at the 3- and 6-month follow-ups.

**Discussion:**

Both FSAK and TIOL implantation effectively reduce pre-existing moderate astigmatism in patients with cataract. The eyes with bifocal TIOLs had more stable long-term astigmatism correction, whereas those with trifocal IOLs and FSAK had better intermediate VA. Therefore, a mix-and-match implantation of trifocal IOL with FSAK and contralateral bifocal TIOL could achieve effective astigmatism correction and provide an overall optimal VA.

## 1. Introduction

Astigmatism correction results in significant vision improvements, such as higher reading performances and uncorrected distance visual acuity (UDVA) (1, 2). The correction of preoperative corneal astigmatism (CA) combined with the implantation of intraocular lenses (IOLs) plays an important role in modern cataract surgery. However, implants with multifocal IOLs are typically not recommended for patients with cataract and a CA > 0.75 D (3), since residual astigmatism (RsA) could undermine the postoperative visual performance, reducing the patient’s satisfaction (4, 5). Therefore, minimizing the postoperative RA is the key to reach an optimal visual acuity (VA) and satisfactory quality of vision in this population.

Over the past several decades, various approaches have been proposed to reduce the pre-existing astigmatism during cataract surgery, including limbal relaxing incisions (LRIs), clear corneal incision on the steep axis (CCI), toric intraocular lens (TIOL) implantation, intracorneal ring segments (ICRSs) and excimer laser refractive procedure (6–8). However, CCI has limitations on the incision location and can only correct mild-to-moderate CA (9, 10); the efficacy of LRI is inconsistent (11–13); ICRs may lead to extrusion, microbial keratitis and corneal meltingnity, and excimer laser refractive procedures are expensive and require a second intervention (14).

Among these procedures, TIOL implantation and femtosecond laser-assisted arcuate keratotomy (FSAK) are the options most frequently used to treat the pre-existing astigmatism accurately. In particular, the implantation of bifocal TIOLs in cataract surgery allows effective management of astigmatism and provides optimal UDVA, uncorrected near visual acuity (UNVA), and overall quality of vision (15–17). The only downside of this procedure is the lack of clear vision at a middle distance. To compensate for the intermediate VA, a trifocal TIOL is more suitable for the patients with cataract and CA who are having high demands of whole-course visual acuity because of its three foci design and astigmatism correction ability. It is a great pity that trifocal TIOLs had not been approved in China at the time of the study, and even now they are not widely available in many regions. Alternatively, FSAK has been proved to provide high precision, safety, and reproducibility in reducing CA and has been suggested as an alternative to TIOL implantation (13, 18). Previous studies reported no significant difference in postoperative RsA between FSAK and conventional cataract surgery with toric monofocal IOL implantation (19, 20). Therefore, FSAK combined with trifocal IOL implantation may be a feasible solution to compensate for the intermediate VA and improve the overall VA in patients ineligible for trifocal TIOLs or living in the areas lack of trifocal TIOLs.

To the best of our knowledge, this study was the first study to compare the efficacy in astigmatism correction and the postoperative visual outcomes between two groups of eyes in patients undergoing a mix-and-match procedure, comprising trifocal IOL implantation combined with FSAK in one eye and bifocal TIOL implantation in the other eye. We aimed to explore the possible advantages of this treatment design for overall VA and possibly also provide a new option for refraction correction in patients with moderate astigmatism.

## 2. Materials and methods

### 2.1. Study design and participants

**Figure 1:**
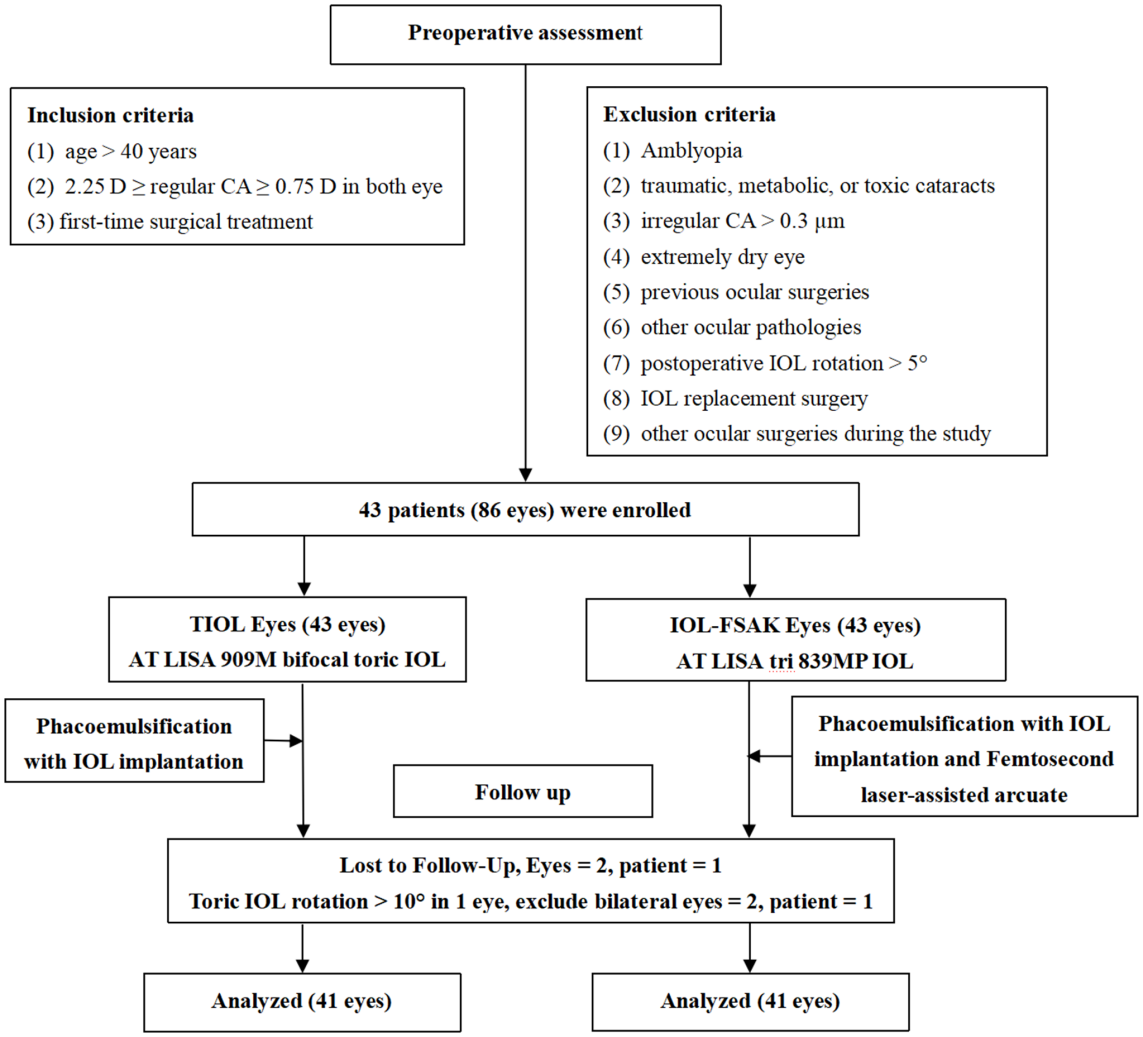
Flowchart of patients enrollment and follow-up.

This prospective observational paired-eye study included adult patients with cataract and *CA.* The patients were hospitalized for cataract surgery and implanted with AT LISA 909 M bifocal TIOLs (Carl Zeiss Meditec, Jena, Germany) in one eye and AT LISA tri 839MP trifocal IOLs (Carl Zeiss Meditec) with simultaneous FSAK in the other eye. Figure 1 shows the flowchart. The characteristics of the lenses are presented in Supplementary Table 1 (21–24).

The procedures were performed between June 2020 and August 2022 at the Shanghai Heping Eye Hospital and the Eye and Ear, Nose, and Throat (ENT) Hospital of Fudan University in Shanghai. The inclusion criteria were as follows: (1) age > 40 years, (2) preoperative regular CA between 0.75 and 2.25 D in both eyes, and (3) first-time surgical treatment. The exclusion criteria were the following: (1) amblyopia; (2) traumatic, metabolic, or toxic cataracts; (3) irregular CA > 0.3 μm; (4) extremely dry eye; (5) previous ocular surgeries; (6) other ocular pathologies, such as diabetic retinopathy, macular degeneration, or glaucoma with field defects; and (7) postoperative IOL rotation >5°, IOL replacement surgery, or other ocular surgeries during the study. The inclusion and exclusion criteria were applied based on the diagnosis, medical history, progress notes, and other medical records throughout the study period. All patients had a follow-up period of at least 6 months. The study was approved by the Institutional Review Board of the Eye and ENT Hospital of Fudan University and the Shanghai Heping Eye Hospital. All procedures adhered to the tenets of the Declaration of Helsinki, and written informed consent was obtained from all patients.

### 2.2. Pre-and postoperative examinations

Each patient underwent a complete preoperative eye examination, including measurements of UDVA at 5 m, best corrected VA (BCVA) at 5 m, and UNVA at 40 cm, using the logarithm of the minimum angle of resolution (logMAR) acuity charts under photopic conditions. Slit-lamp biomicroscopy, fundoscopy, and intraocular pressure measurement were also performed. Other preoperative evaluations included subjective refraction, optical biometry with IOL-Master 700 (Carl Zeiss Meditec), higher-order aberrations (HOAs), Strehl ratio (SR), and modulation transfer function (MTF) curve with the HOYA iTrace ray-tracing system (Tracey Technologies, Houston, TX, United States). Keratometry was performed using corneal topography (Pentacam, OCULUS Optikgeraete GmbH, Wetzlar, Germany), and the values obtained were used to determine the arcuate keratotomy profile and analyze the postoperative outcomes. Postoperative examinations, including UDVA, BCVA, uncorrected intermediate visual acuity (UIVA) at 80 cm, UNVA, IOL rotation, subjective refraction, and CA, were performed 1 week after each surgery (with 1-week intervals between them) and afterwards at 1, 3, and 6 months. The HOAs, SR, MTF curve and defocus curve were measured 3 months after the surgery. In addition, all the side effects or complications that occurred during the 6-month follow-up period were recorded.

### 2.3. Surgical technique

The first surgical eye in each patient was defined as the eye with worse BCVA; if both eyes had the same BCVA, the right eye was selected as the first surgical eye. This eye underwent a conventional phacoemulsification surgery with implantation of a 909 M bifocal TIOL (henceforth named TIOL eye). The other eye was operated with FSAK using a Lensx laser system (Alcon Vision LLC, Fort Worth, TX, United States) and implanted with an 839MP trifocal IOL (IOL-FSAK eye). Both the phacoemulsification surgery and FSAK were performed by Dr. J. Y. using a standardized surgical technique under surface anesthesia. Horizontal limbal marking was performed in all patients in a sitting position prior to the surgery.

In the TIOL eyes, a 2.2 mm transparent corneal incision at 140° and a 5.4 mm central continuous circular capsulorhexis were performed under the guidance of the CALLISTO eye intraoperative navigation system (Carl Zeiss Meditec). After a standard divide-and-conquer phacoemulsification technique, the bifocal TIOL was implanted into the capsular bag. The IOL axial alignment was adjusted to the implant axis, and the incision watertightness was confirmed.

In the IOL-FSAK eyes, capsulotomy and lens fragmentation were performed with a laser and followed by arcuate keratotomy and creation of a 2.2 mm main incision at 140°. The arc length and axis of incisions were calculated using an online calculator.^1^ Briefly, all arcuate keratotomies comprised a pair of symmetric intrastromal incisions with a depth of 90% of the corneal thickness and an optical zone diameter of 8.5 mm. After the laser-assisted process, the fragmented lens was removed with a standard phacoemulsification, as in the first eye, and the trifocal IOL was implanted.

The IOL power and axial alignment were calculated using the IOL Master 700 and Barrett Toric formulas, with the surgically induced astigmatism (SIA) set to 0.2 D and the target refraction set to 0.0 D. As the CA changes from with-the-rule (WTR) to against-the-rule (ATR) with age (25–27), the postoperative RsA was selected as the WTR astigmatism closest to 0.0 D.

### 2.4. Statistical analysis

All statistical analyses were performed using SPSS Statistics version 26.0 (IBM, Armonk, NY, USA). The quantitative variables are expressed as mean ± standard deviation (SD), whereas the qualitative variables as absolute numbers (n) and frequencies (%). Paired t-tests were performed on continuous data to assess the differences between groups and between pre-and postoperative values within the same group and *χ*^2^ tests to compare the categorical data. Group sample sizes of 41 can achieve 79.9% power to reject the null hypothesis of zero effect size when the population effect size is 0.50 and the significance level (alpha) is 0.05 using a two-sided two-sample equal-variance *t*-test. Statistical significance was set at *p* < 0.05.

## 3. Results

### 3.1. Baseline characteristics

Initially, 43 patients (86 eyes) were enrolled in this study. However, one patient was lost to follow-up, and one was excluded owing to toric IOL rotation >10°, with consequent IOL replacing surgery 2 weeks after the first one. Therefore, 82 eyes from 41 patients (19 men and 22 women; age range: 29–76 years, mean: 53.81 ± 13.28) were finally included in the analysis. The baseline findings of the two eye groups are presented in Table 1. There were no statistically significant differences between the two groups in baseline clinical indicators.

**Table 1:** Demographics and clinical characteristics of the two eye groups.

Parameter	Overall	Trifocal IOL with FSAK	Bifocal TIOL	*p* value
Number of eyes, *n*	82	41	41	
Age, median (range), years	53.5 (29–76)			
Sex, males/females, *n*	19/22			
ATR/WTR/Oblique	12/61/9	6/30/5	6/31/4	
Axial length, mm	26.30 ± 2.26	26.18 ± 2.32	26.42 ± 2.24	0.48
ACD, mm	3.36 ± 0.43	3.37 ± 0.41	3.35 ± 0.45	0.36
Lens thickness, mm	4.20 ± 0.46	4.18 ± 0.49	4.22 ± 0.44	0.52
White-to-White, mm	11.72 ± 0.56	11.72 ± 0.56	11.72 ± 0.57	0.34
Corneal astigmatism, D	1.51 ± 0.42	1.46 ± 0.39	1.57 ± 0.44	0.24
Preoperative UDVA, LogMAR	0.96 ± 0.38	0.99 ± 0.43	0.93 ± 0.33	0.51
Preoperative UNVA, LogMAR	0.39 ± 0.32	0.37 ± 0.31	0.40 ± 0.33	0.62
Preoperative CDVA, Logar	0.40 ± 0.38	0.37 ± 0.35	0.42 ± 0.42	0.61
Predicted SE, D	−0.19 ± 0.17	−0.18 ± 0.16	−0.21 ± 0.18	0.66
Predicted RA, D	−0.12 ± 0.11	−0.10 ± 0.08	−0.13 ± 0.13	0.47

The data are expressed as mean ± standard deviation, unless indicated otherwise. ACD, anterior chamber depth; ATR, against-the-rule; WTR, with-the-rule; CDVA, corrected distance visual acuity; RA, residual astigmatism; SE, spherical equivalent; UDVA, uncorrected distance visual acuity; UNVA, uncorrected near visual acuity.

### 3.2. Visual outcomes and defocus curve

**Figure 2:**
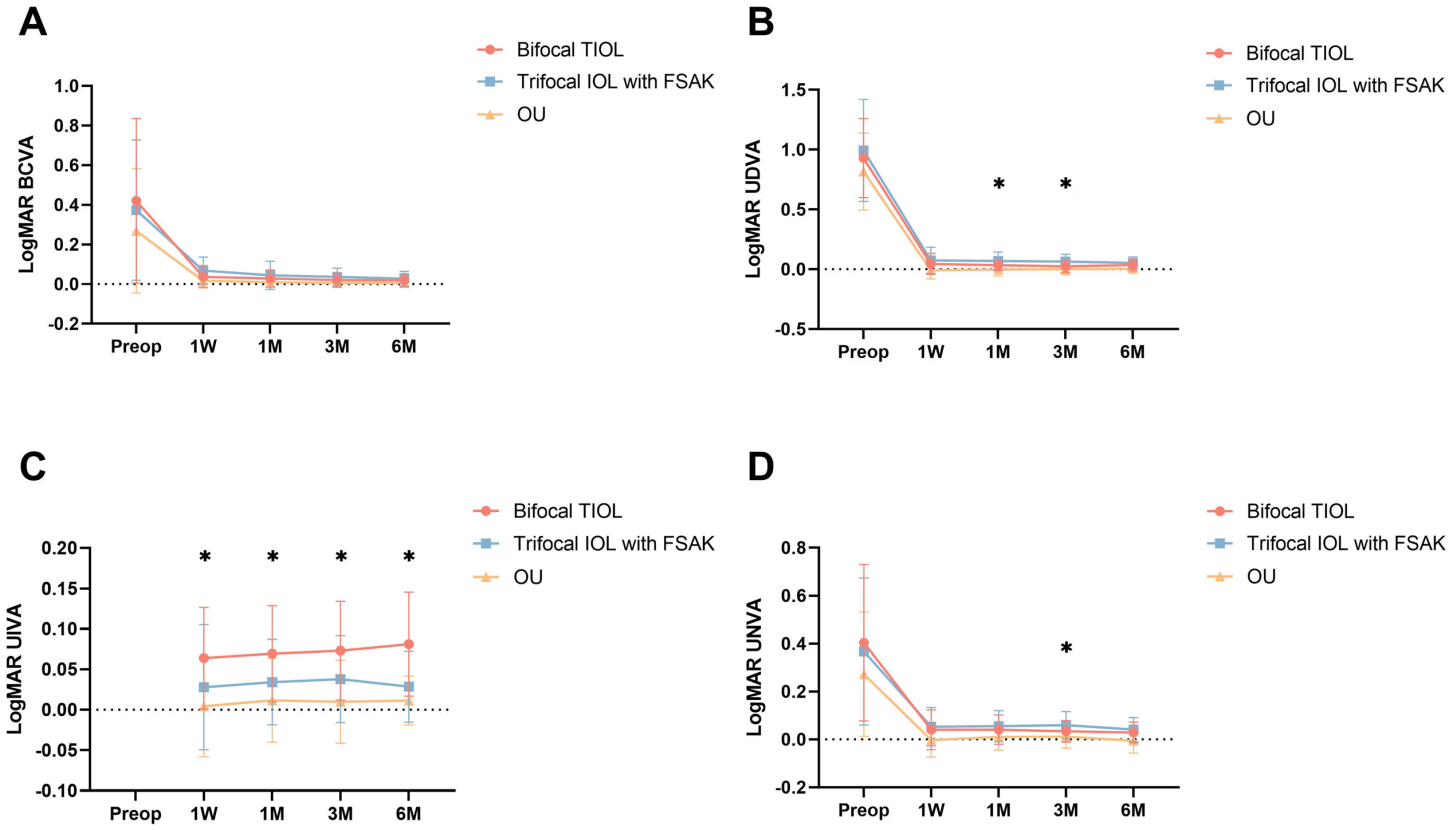
Baseline and postoperative visual outcomes of the two groups at 1 week and 1, 3, and 6 months postoperatively. All data are presented as mean ± standard deviation. **(A)** BCVA (logMAR); **(B)** UDVA (logMAR); **(C)** UIVA (logMAR); **(D)** UNVA (logMAR). OU, binocular vision. *p < 0.05.

Figure 2 presents the postoperative visual acuity of the two groups at every visit. Each patient obtained satisfying binocular visual acuity after surgery. Significant inter-group differences were observed in UIVA at 1 week and 1, 3, and 6 months postoperatively. The TIOL eyes displayed greater 1- and 3-month UDVA (1 month: 0.03 ± 0.05 vs. 0.08 ± 0.08 logMAR, *p* = 0.036; 3 months: 0.02 ± 0.05 vs. 0.06 ± 0.06 logMAR, *p* = 0.010) and greater 3-month UNVA (0.03 ± 0.04 vs. 0.06 ± 0.06 logMAR, *p* = 0.030) than did the IOL-FSAK eyes. No other significant differences in UDVA/BCVA/UNVA emerged at other follow-up points between the two groups.

**Figure 3:**
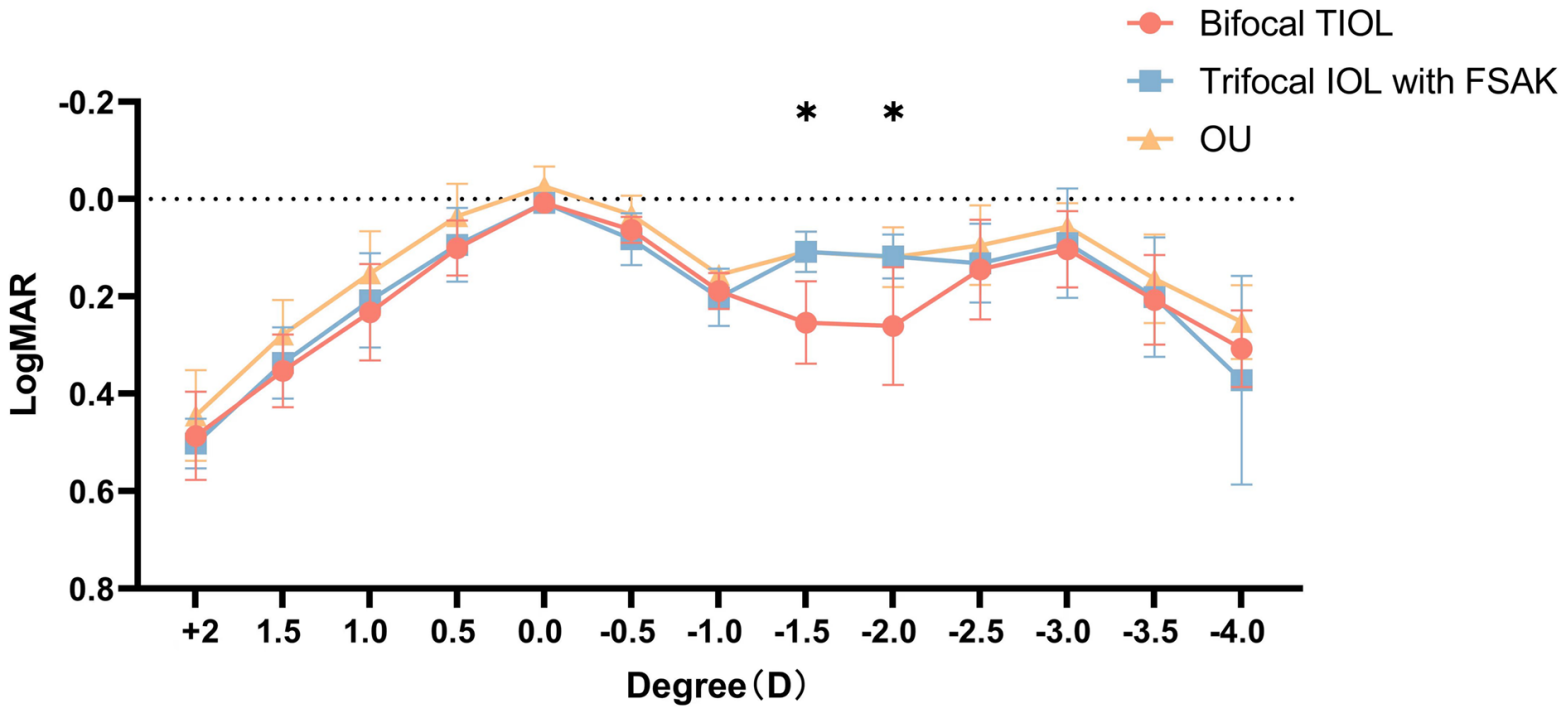
Monocular defocus curves of bifocal TIOL eyes and trifocal IOL with FSAK eyes, and binocular (OU) defocus curves of all participants. All data are presented as mean ± standard deviation. *p < 0.05.

Defocus curves (Figure 3) were available for 35 patients, both in each eye and bilaterally. The IOL-FSAK eyes showed a significantly greater VA than did the TIOL eyes in the intermediate range (−1.50 D: 0.11 ± 0.04 vs. 0.25 ± 0.08 logMAR, *p* = 0.027; −2.00 D: 0.12 ± 0.05 vs. 0.26 ± 0.12 logMAR, *p* = 0.047). No other significant differences were noted between the two eye groups at other focuses.

### 3.3. Postoperative refractive astigmatism and corneal astigmatism

**Figure 4:**
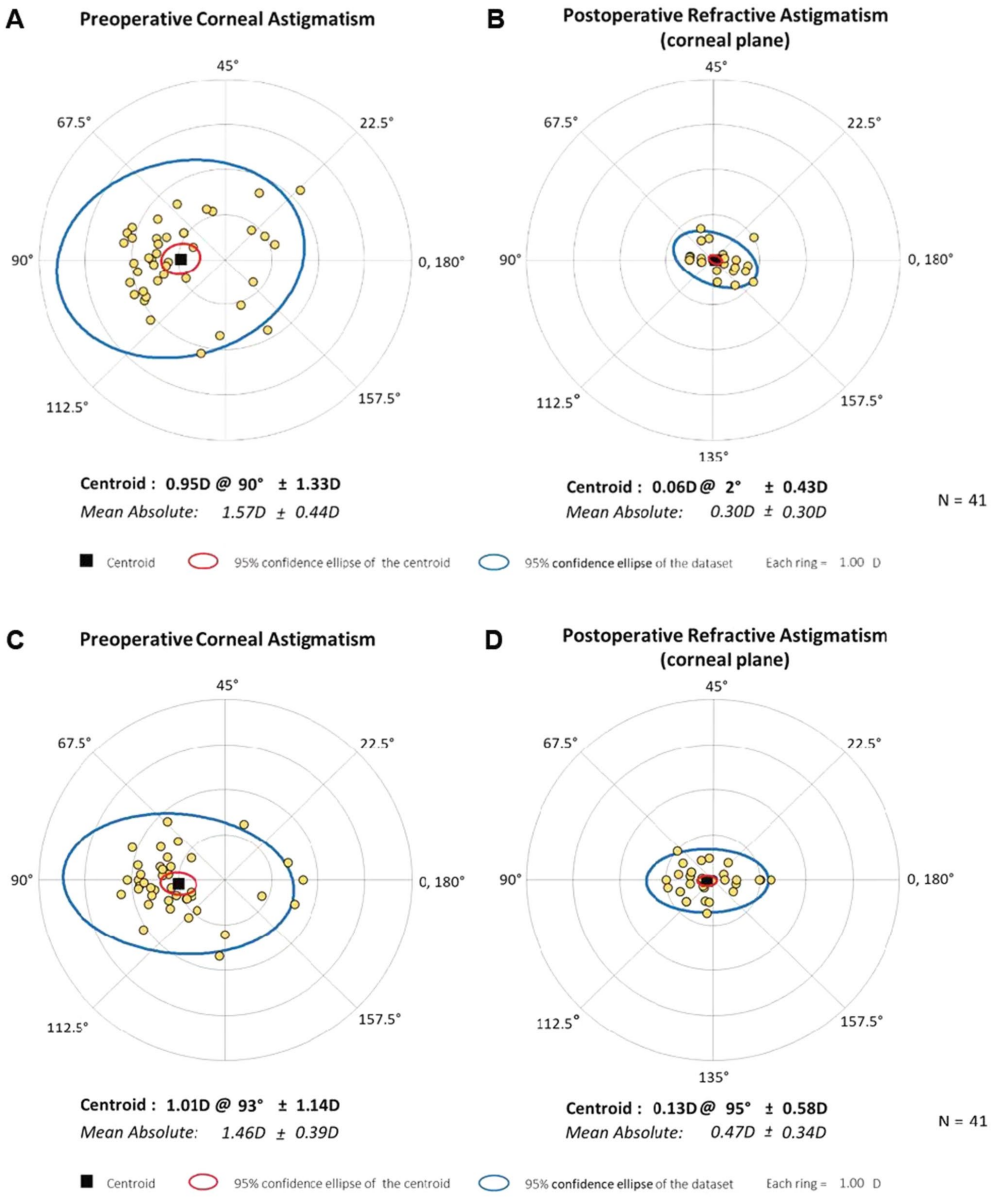
Double-angle plots of preoperative and 6-month postoperative astigmatism in the two eye groups. **(A)** Preoperative corneal astigmatism of bifocal TIOL eyes; **(B)** Postoperative refractive astigmatism in spectacle plane of bifocal TIOL eyes at 6 months; **(C)** Preoperative corneal astigmatism of trifocal IOL with FSAK eyes; **(D)** Postoperative refractive astigmatism in spectacle plane of trifocal IOL with FSAK eyes at 6 months. Each ring represents 1.0 D.

**Figure 5:**
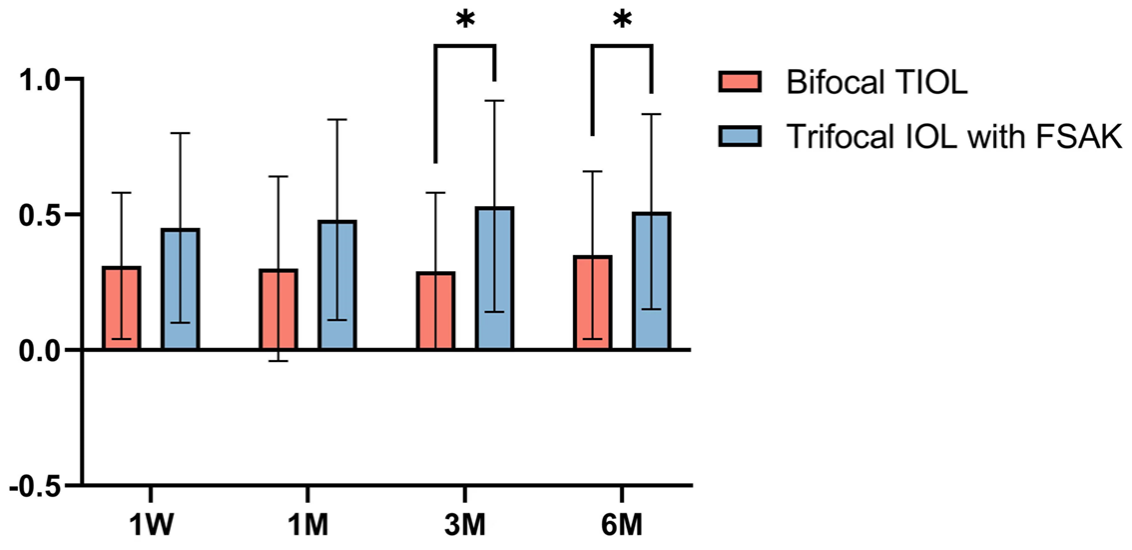
Box-whisker plots of pre-and postoperative refractive astigmatism in the two groups at each follow-up point. All data are presented as a mean ± standard deviation. *p < 0.05.

The double-angle plots (Figure 4) show the RfA and CA of the two groups at baseline and 6-month postoperatively; more details are presented in Table 2. The postoperative mean RfA was significantly lower than the baseline in both groups (all *p* < 0.05). Moreover, the postoperative CA of the IOL-FSAK eyes was markedly lower than baseline at every visit, whereas the postoperative CA of the TIOL eyes slightly increased. Furthermore, there was a significant difference in CA between subsequent time points in the IOL-FSAK eyes (0.54 ± 0.28 D, 0.61 ± 0.33 D, 0.63 ± 0.27 D, and 0.72 ± 0.23 D at 1 week and 1, 3, and 6 months postoperatively, respectively; *p* < 0.001). A significant difference in postoperative CA was observed between the two groups at every follow-up. Finally, the postoperative RA in the IOL-FSAK eyes was significantly higher than that in the TIOL eyes at 3- and 6-month follow-up visits (Figure 5).

**Table 2:** Corneal and refractive astigmatism in the two eye groups at each follow-up time point.

	Trifocal IOL with FSAK (*n* = 41)	Bifocal TIOL (*n* = 41)	P^1^	*P* ^2^	*P* ^3^
Manifest refractive astigmatism, D					
Baseline	−0.97 ± 0.64	−1.79 ± 1.03			
1-week postoperative	−0.45 ± 0.35	−0.31 ± 0.27	0.021*	< 0.001*	0.110
1-month postoperative	−0.48 ± 0.37	−0.30 ± 0.34	0.008*	< 0.001*	0.122
3-month postoperative	−0.53 ± 0.39	−0.29 ± 0.29	0.012*	< 0.001*	0.032*
6-month postoperative	−0.51 ± 0.36	−0.35 ± 0.31	0.005*	< 0.001*	0.036*
Corneal astigmatism, D					
Baseline	1.46 ± 0.39	1.57 ± 0.44			
1-week postoperative	0.54 ± 0.28	1.69 ± 0.56	< 0.001*	0.289	0.001*
1-month postoperative	0.61 ± 0.33	1.68 ± 0.60	< 0.001*	0.339	0.003*
3-month postoperative	0.63 ± 0.27	1.73 ± 0.57	< 0.001*	0.564	0.003*
6-month postoperative	0.72 ± 0.23	1.71 ± 0.56	< 0.001*	0.364	0.007*
Mean percentage of astigmatic reduction (%) from baseline					
1-week postoperative	53.41%	6.89%			
1-month postoperative	46.97%	7.06%			
3-month postoperative	44.76%	3.34%			
6-month postoperative	36.16%	5.26%			

The data are expressed as mean ± standard deviation or percentages as appropriate. *P*^1^: *p*-value of the differences with baseline in the IOL-FSAK group. *P*^2:^ *p*-value of the differences with baseline in the TIOL group. *P*^3^: *p*-value of the differences between the groups. *significant differences.

### 3.4. High-order aberrations and objective visual quality

Three months after the surgery, similar results were observed for total objective visual quality in the two groups (Table 3). No significant differences in total HOAs, MTF-10, MTF-30, and SR were noted between the TIOL and IOL-FSAK eyes. However, the corneal visual quality values were significantly higher in the IOL-FSAK eyes than in the TIOL eyes (corneal HOAs: 0.083 ± 0.048 vs. 0.082 ± 0.035, *p* = 0.032; corneal MTF-10: 0.429 ± 0.224 vs. 0.194 ± 0.142, *p* = 0.003; corneal MTF-30: 0.120 ± 0.102 vs. 0.047 ± 0.037, *p* = 0.008; corneal SR: 0.170 ± 0.161 vs. 0.057 ± 0.053, *p* = 0.015).

**Table 3:** Postoperative high-order aberrations in the two groups at 3 months after surgery in 52 eyes of 26 patients.

	Overall (*n* = 52)	Trifocal IOL with FSAK (*n* = 26)	Bifocal TIOL (*n* = 26)	*p*-value
HOAs total, μ	0.12 ± 0.07	0.12 ± 0.07	0.12 ± 0.07	0.064
Coma, μ	0.05 ± 0.05	0.05 ± 0.06	0.06 ± 0.04	0.405
Spherical, μ	0.01 ± 0.02	0.01 ± 0.02	0.02 ± 0.03	0.318
Trefoil, μ	0.07 ± 0.04	0.07 ± 0.05	0.06 ± 0.03	0.136
Secondary astigmatism, μ	0.03 ± 0.03	0.03 ± 0.04	0.03 ± 0.03	0.319
HOAs corneal, μ	0.08 ± 0.04	0.08 ± 0.04	0.08 ± 0.05	0.032*
MTF-10 total	0.42 ± 0.19	0.41 ± 0.19	0.43 ± 0.20	0.112
MTF-10 corneal	0.31 ± 0.22	0.43 ± 0.22	0.19 ± 0.14	0.003*
MTF-30 total	0.14 ± 0.09	0.14 ± 0.11	0.14 ± 0.08	0.399
MTF-30 corneal	0.08 ± 0.08	0.12 ± 0.10	0.05 ± 0.04	0.008*
SR total	0.20 ± 0.17	0.20 ± 0.19	0.19 ± 0.15	0.448
SR corneal	0.11 ± 0.13	0.17 ± 0.16	0.06 ± 0.05	0.015*

The data are expressed as mean ± standard deviation. HOAs, high-order aberrations; MTF, modulation transfer function; SR, Strehl ratio. *significant differences.

### 3.5. Postoperative complications

Only one young patient (29 years) incurred mild posterior capsule opacity (PCO) in both eyes; no other serious complications, such as endophthalmitis, corneal decompensation, or retinal detachment, occurred during the follow-up period. The patient with mild PCO had no significant repercussions on the postoperative VA and did not require yttrium aluminum garnet (YAG) laser posterior capsulotomy; therefore, he was not excluded from the study.

## 4. Discussion

Several studies examined the efficacy of FSAK and TIOL in correcting astigmatism; however, which technique is the most effective remains to be determined. Moreover, these studies were usually based on small cohorts of patients, and the postoperative effect of astigmatism correction was determined through different patients undergoing different procedures, likely incurring in selection bias and individual result differences. To the best of our knowledge, this study is the first to evaluate the postoperative visual outcomes by using two astigmatism correction methods simultaneously, one per eye, in the same patient. This paired-eye study offers more reliable results and more effective comparisons than single-method studies and provides a new technical option for refractive cataract surgery accompanied by astigmatism correction in clinical practice.

The patients enrolled in this study had mild or moderate CA (<2.25 D). In previous FSAK studies, the keratometric astigmatic changes ranged from 0.69 D to 1.21 D, and the percentages of astigmatic reduction ranged from 38.8 to 58% (17, 28, 29). The astigmatism correction capability is determined by the incisional depth and optical zone diameter (30, 31). According to a study by Wang et al., FSAK can achieve a reduction of 46.1–47.5% of the preoperative CA with an incisional depth of 90% and optical zone diameter of 8.0 mm (32). Therefore, we set the incisional depth at 90% and the optical zone diameter at 8.5 mm and selected patients with preoperative astigmatism <2.25 D to obtain a postoperative astigmatism within 1.0 D.

Based on the results of the postoperative RfA, the preoperative CA was corrected effectively in all patients, with no RsA > 1.0 D. The TIOL eyes had a lower postoperative RsA, ≤ 0.5 D in 35/41 eyes (85.37%), than 28/41 (68.29%) of IOL-FSAK eyes. In addition, the mean postoperative RsA in the IOL-FSAK eyes was higher than that in the TIOL eyes at 3 and 6 months after surgery, indicating that the long-term efficacy and stability of astigmatism correction in the TIOL eyes were more consistent than those in the IOL-FSAK eyes. In terms of CA correction, significant reductions were noted in the IOL-FSAK eyes at each postoperative visit. However, the correction regressed gradually over time, and the percentage of astigmatism reduction decreased from 53% at 1 week to 36% at 6 months postoperatively, in line with the results reported in previous studies (13, 17). In contrast, the TIOL eyes had a mean increase in CA of 0.12 D from baseline to 1 week after surgery and of 0.16 D at the 3-month follow-up. Most patients in this study had WTR and oblique astigmatism, and a 140° clear corneal incision may lead to variable surgically induced astigmatism; this effect explains the increased CA in the early postoperative period, which remained stable in the 6-month follow-up (33).

In terms of VA, the TIOL eyes were more stable than the IOL-FSAK eyes. The postoperative VA in the TIOL eyes was consistently high, except in the case of marked intraocular rotation. The IOL-FSAK eyes, on the other hand, showed fluctuations in VA, especially the UDVA. The astigmatic correction of the trifocal IOL with the FSAK procedure met the expectations at 1 week postoperatively and remained stable in the first month, with optimal VA; however, with the regression of the astigmatism correction, the postoperative RsA gradually increased 3 and 6 months after surgery, manifesting as decreased UDVA. This finding is consistent with that of a previous study, showing that when the RsA is elevated, the UDVA is affected first, and the patients complain of a decrease in distant VA (34). Afterward, if the RsA continues to increase, the UNVA is also affected, with a decline in near VA noticeable in daily life as reduced discrimination ability for fine objects and small prints. However, this study showed an interesting phenomenon: despite the decreasing astigmatism correction in the IOL-FSAK eyes, lower than that in the TIOL eyes at 6 months, the differences in UDVA and UNVA between the two groups were not significant. We speculate that the tolerance to astigmatism in the visual center of the brain may increase over time owing to neural adaptation, allowing the patients to tolerate low-degree RsA after surgery and resulting in clearer vision (35).

Another interesting finding was that 3 months after surgery, the objective visual quality of total eye, quantified by total HOAs, SR, and MTF curves (MTF-10 and MTF-30), showed high retinal optical qualities of both lenses but no significant difference between the groups (36). Thus, FSAK effectively controlled the postoperative RA despite being less effective than TIOL in correcting astigmatism, and the postoperative visual quality was not significantly affected. Therefore, FSAK is an effective, repeatable, and accurate method for astigmatism correction. It may also be a good alternative for patients with mild or moderate CA to achieve an optimal overall vision without the use of a trifocal TIOL. Furthermore, most patients reported that the intermediate VA was greater in the IOL-FSAK eye. This effect was confirmed in the postoperative statistics of UIVA and defocus curves, particularly the binocular defocus curve, which reflects the actual visual quality in patients performing intermediate-distance activities in daily life. Based on the binocular defocus curve, these patients had excellent visual continuity in the whole visual range, without obvious troughs, suggesting that the mix-and-match procedure design could compensate for the deficiencies in intermediate-distance VA, providing a clear overall VA.

The limitations of this study included the relatively small sample size and the short duration of the follow-up. The former may lead to statistical bias, which could be eliminated in future large-scale, multi-center studies, whereas a longer follow-up time will allow more thorough observations of CA changes. In addition, this study was designed to compare the procedures in two eyes from the same patient; therefore, subjective quality assessment of the two eyes separately could not be obtained, and the influence of RsA on the degree of postoperative photic phenomena could not be compared. Moreover, although a strict paired study should implant the same IOL in both eyes, with only different corneal astigmatic correction methods, this study chose mix-and-match implantation of bifocal TIOL and trifocal IOL to improve the overall VA of patients.

In conclusion, FSAK could be an effective, reproducible, and precise procedure to reduce pre-existing CA in cataract surgery combined with IOL implantation. The stability and effectiveness of the bifocal TIOLs were higher than those of trifocal IOLs and FSAK in the long-term follow-up; however, for patients with mild or moderate CA, a mix-and-match procedure comprising trifocal IOL implantation and FSAK in one eye and bifocal TIOL implantation in the other could provide greater overall VA and quality of vision than could either procedure in both eyes.

## Data availability statement

The raw data supporting the conclusions of this article will be made available by the authors, without undue reservation.

## Ethics statement

The studies involving human participants were reviewed and approved by the Institutional Review Board of the Eye and ENT Hospital of Fudan University and the Shanghai Heping Eye Hospital. The patients/participants provided their written informed consent to participate in this study.

## Author contributions

JY and HG designed the research. JS and LZ collected the data. ZH and JS analyzed the data. JS wrote the manuscript. JY and HG critically revised the manuscript. JS, LZ, JY, HG, and ZH contributed equally to this work. All authors contributed to the article and approved the submitted version.

## Funding

This work was funded by the Chinese National Nature Science Foundation (grant no. 82171039) and The Scientific Research Fund of Shanghai Municipal Health and Family Planning Commission (grant no. 201840239).

## Conflict of interest

The authors declare that the research was conducted in the absence of any commercial or financial relationships that could be construed as a potential conflict of interest.

## Publisher’s note

All claims expressed in this article are solely those of the authors and do not necessarily represent those of their affiliated organizations, or those of the publisher, the editors and the reviewers. Any product that may be evaluated in this article, or claim that may be made by its manufacturer, is not guaranteed or endorsed by the publisher.

## Footnotes

^1^ https://www.lricalculator.com/
