# Supplementary material for: Comparison of astigmatism correction and visual outcomes in mix-and-match implantations of trifocal intraocular lenses with femtosecond laser-assisted arcuate keratotomy and contralateral bifocal Toric intraocular lenses
Source: Front Med (Lausanne). 2023 Aug 4;10:1237319. doi: 10.3389/fmed.2023.1237319 (PMC10436569; doi:10.3389/fmed.2023.1237319)
Supplement: Supplementary file 1 [file Table_1.DOCX]

Supplementary Material

Comparison of Astigmatism Correction and Visual Outcomes in Mix-and-Match Implantations of Trifocal Intraocular Lenses with Femtosecond Laser-Assisted Arcuate Keratotomy and Contralateral Bifocal Toric Intraocular Lenses

Jiying Shen, Zhixiang Hua, Wenqian Shen, Limei Zhang, Haike Guo, Jin Yang

*** Correspondence:** Jin Yang and Haike Guo: jin_er76@hotmail.com (JY); guohaike@hotmail.com (HKG)

# Supplementary Table

Supplementary Table 1. Properties of the two IOLs used in the present study.

| Name | AT LISA tri 839 MP | AT LISA 909M TIOL |
| --- | --- | --- |
| Optics | Diffractive | Diffractive |
| Material | Hydrophilic acrylic | Hydrophilic acrylic |
| Near add, D | +3.33 | +3.66 |
| Intermediate add, D | +1.66 | None |
| Diffractive steps | 21-29 | 15 |
| Edge design | 360° square edge | 360° square edge |
| Light transmission | 86% | 88% |
| Light distribution |  |  |
| Far | 50% | 65% |
| Intermediate | 20% | None |
| Near | 30% | 35% |
| Optic diameter, mm | 6.0 | 6.0 |
| Overall diameter, mm | 11.0 | 11.0 |
